# Structural insights into histone mimicry by the small hepatitis delta antigen

**DOI:** 10.1016/j.jbc.2026.113252

**Published:** 2026-06-12

**Authors:** Haiyun Hu, Mengjiao Lv, Xiaohui Wang, Xinci Shang, Qingyan Wu, Yichang Chen, Yinhao Zhou, Qiuyan Huang, Tao Jiang, Su Qin, Xiaolei Huang, Zhenfeng Zhang, Guoqiang Xu, Yanli Liu

**Affiliations:** 1 Jiangsu Key Laboratory of Drug Discovery and Translational Research for Brain Diseases, College of Pharmaceutical Sciences, Soochow University, Suzhou, Jiangsu, China; 2 Jiangsu Province Engineering Research Center of Precision Diagnostics and Therapeutics Development, Soochow University, Suzhou, Jiangsu, China; 3 Suzhou International Joint Laboratory for Diagnosis and Treatment of Brain Diseases, Suzhou, Jiangsu, China; 4 School of Public Health and Emergency Management, School of Medicine, Southern University of Science and Technology, Shenzhen, Guangdong, China; 5 Life Science Research Center, Southern University of Science and Technology, Shenzhen, Guangdong, China; 6 Biomedical Basic Research Center (BBRC) of Jiangsu Province, Suzhou Medical College of Soochow University, Suzhou, Jiangsu, China; 7 Suzhou Key Laboratory of Geriatric Neurological Disorders, the First People’s Hospital of Taicang, Taicang Affiliated Hospital of Soochow University, Suzhou, Jiangsu, China

**Keywords:** HDV, S-HDAg-K72ac, BAZ2 protein, bromodomain, complex structure

## Abstract

Hepatitis delta virus (HDV) is a satellite RNA virus that requires hepatitis B virus (HBV) for propagation but replicates its genome independently in the nucleus. The small form of the hepatitis delta antigen (S-HDAg) is essential for replication and is regulated by post-translational modifications. Acetylation at lysine 72 (K72ac) enables S-HDAg to interact with the bromodomain (BRD) of the host chromatin remodeler bromodomain adjacent to zinc finger domain protein 2B (BAZ2B) to promote viral replication. However, the structural basis for this interaction has remained elusive. Here, we provide structural and biophysical insights into this interaction through quantitative binding assays and X-ray crystallography. Isothermal titration calorimetry revealed that BRDs of BAZ2B and its close homolog BAZ2A bind to the viral peptide weakly, with BAZ2A-BRD exhibiting a modestly higher affinity. The crystal structure of BAZ2A-BRD in complex with the S-HDAg-K72ac peptide demonstrates an inverted binding orientation relative to canonical histone ligands, rationalizing the weak interaction. Mutagenesis studies confirmed the critical binding interface both *in vitro* and in cells. These findings elucidate the molecular mechanism by which HDV co-opts host BAZ2 bromodomains *via* a unique, weak-affinity interaction, providing a structural framework for understanding viral replication.

Hepatitis delta virus (HDV) is a negative-sense, single-stranded RNA virus with a 1.7 kb circular genome (1). As a satellite virus of hepatitis B virus (HBV), HDV depends on HBV to supply envelope proteins for virion assembly and infectivity (1). HDV infection affects approximately 8.7 to 18.7 million people worldwide and represents the most severe form of viral hepatitis, for which limited effective antiviral therapy currently exists (2, 3). Understanding the molecular mechanisms by which HDV hijacks host cellular machinery is therefore of high clinical relevance. However, HDV genome replication, which occurs *via* a double-rolling-circle mechanism in the nucleus, is independent of HBV (4, 5, 6, 7). The HDV genome encodes only one viral protein, the hepatitis delta antigen (HDAg), which exists in two forms: Large HDAg (L-HDAg, 214 amino acids for genotype 1) and small HDAg (S-HDAg, 195 amino acids) (Fig. 1*A*) (8, 9, 10). The two HDAg proteins are identical except for a 19 or 20-amino-acid C-terminal extension present in L-HDAg, resulting from RNA editing of the stop codon of S-HDAg (8, 9, 10). Both proteins contain a coiled-coil dimerization domain (CDD), a nuclear localization signal (NLS), and two RNA-binding domains (RBDs) within their common region, and L-HDAg additionally carries a unique nuclear export signal (NES) in its C-terminal extension (11, 12, 13, 14, 15, 16). Despite being highly similar in sequence, the two forms of HDAg play distinct roles in the HDV life cycle: L-HDAg is required for virion assembly (17), whereas S-HDAg is essential for HDV RNA replication (4, 18). Furthermore, like many host proteins, both HDAgs undergo post-translational modifications that regulate their functions. For instance, isoprenylation at C211 in L-HDAg’s C-terminal extension mediates interaction with the HBV surface antigen to promote HDV virion assembly (19, 20). Meanwhile, acetylation at K72 and methylation at R13 in S-HDAg modulate its subcellular localization and RNA replication (21, 22), and phosphorylation at S177 facilitates the switch from initiation to elongation during antigenomic RNA synthesis (23). Additionally, a recent study showed that acetylated S-HDAg at K72 (S-HDAg-K72ac) interacts with bromodomain adjacent to zinc finger protein 2B (BAZ2B), a regulatory subunit of the imitation switch (ISWI) chromatin remodeling BRF (BAZ2B-associated remodeling factor) complexes (24), thereby recruiting BRF complexes to HDV ribonucleoproteins to sustain viral RNA replication (25).

**Figure 1:**
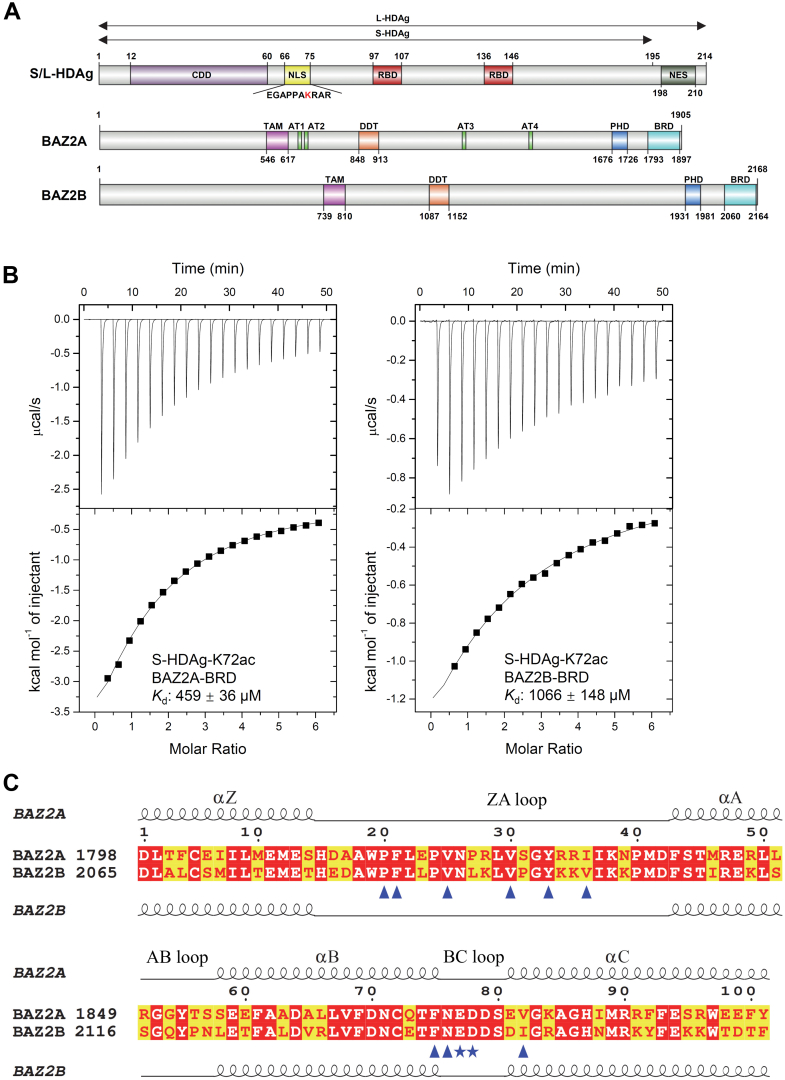
**S-HDAg-K72ac binds to the BRDs of BAZ2A and BAZ2B with high micromolar affinity.***A*, schematic representation of the domain architecture of L-HDAg, S-HDAg, BAZ2A, and BAZ2B. CDD, coiled-coil dimerization domain; NLS, nuclear localization signal; RBD, RNA-binding domain; NES, nuclear export signal; TAM, TIP5/ARBP/MBD; AT, AT-hook DNA-binding motif; DDT, DNA-binding homeobox and different transcription factors domain; PHD, plant homeodomain zinc finger; BRD, bromodomain. The NLS sequence of HDAg is shown with the acetylated K72 highlighted in *red*. *B*, ITC binding curves for the titration of S-HDAg-K72ac (residues 67–77) into BAZ2A-BRD (residues 1796–1899) and BAZ2B-BRD (residues 2054–2168), respectively. ITC data shown are representative of two independent experiments performed by iTC200 microcalorimeter (MicroCal, Inc.). *K*_d_: dissociation constant (μM). *C*, structure-based sequence alignment of the BRDs of BAZ2A and BAZ2B. Secondary structure elements of BAZ2A-BRD and BAZ2B-BRD are indicated above and below the alignment, respectively. The key residues involved in interaction with S-HDAg-K72ac are marked by *blue* triangles; the two negatively charged residues for recognition of the signature arginine residue in the KacXXR motif are marked by *blue* stars. Alignments were constructed with ClustalW (43) and refined with ESPript (44).

BAZ2B and its homolog BAZ2A, also known as TIP5 (TTF-I-interacting protein 5), serve as regulatory subunits of the ISWI chromatin remodeling family. BAZ2B and BAZ2A associate with SNF2L and SNF2H, the two ATPase subunits, forming the ATP-dependent chromatin remodelers BRF and NoRC (nucleolar remodeling complex), respectively (24, 26). These multidomain proteins share approximately 30% sequence identity overall and exhibit similar domain organization, including a TAM (TIP5/ARBP/MBD) domain, a DNA-binding homeobox and different transcription factors (DDT) domain, and a plant homeodomain (PHD) zinc finger followed by a bromodomain (BRD) (Fig. 1*A*) (27). BAZ2A additionally contains four AT-hook DNA-binding motifs. Despite their structural similarity, the two BAZ2 proteins perform distinct nuclear functions: BAZ2A forms the NoRC complex with SNF2H to help maintain genome stability and repress ribosomal DNA (rDNA) transcription (28, 29, 30, 31, 32), whereas BAZ2B regulates neurodevelopment (33, 34), reprograms hematopoietic committed progenitors (35), influences healthy aging (36), and promotes HDV replication (25).

The BRDs of both BAZ2A and BAZ2B recognize histone marks containing a KacXXR motif, specifically H3K14ac and H4K16ac, with the second “X” preferring a hydrophobic or aromatic residue (37, 38, 39). A recent study demonstrated that HDV hijacks BAZ2B to recruit BRF chromatin remodelers *via* the S-HDAg-K72ac site, which mimics the sequence of known histone ligands of the BAZ2B bromodomain (BAZ2B-BRD), thereby facilitating viral replication (25). However, the structural basis for this interaction remains unclear. In this study, we performed quantitative binding assays and X-ray crystallographic analyses of the BRDs of BAZ2B and its close homolog BAZ2A with an S-HDAg-K72ac peptide. Isothermal titration calorimetry (ITC) revealed that both BRDs bind to the S-HDAg-K72ac peptide weakly, with BAZ2A-BRD exhibiting a modestly higher affinity. The crystal structure of BAZ2A-BRD in complex with S-HDAg-K72ac shows an inverse peptide orientation relative to histone ligands of BAZ2-BRD, consistent with the observed weak binding affinity. Mutagenesis studies further validated the key structural findings both *in vitro* and in cells. Our results elucidate the molecular mechanism underlying the interaction between BAZ2-BRD and S-HDAg-K72ac, providing a structural foundation for understanding HDV replication.

## Results and discussion

### The acetylated S-HDAg-K72 peptide binds to the BRDs of BAZ2A and BAZ2B

A recent study identified BAZ2B as an interactor of S-HDAg through affinity-capture coupled with mass spectrometry. Subsequent pull-down assays confirmed a direct interaction between S-HDAg and the BRD of BAZ2B (BAZ2B-BRD). Further investigation revealed that S-HDAg, as well as the ATPase subunits SNF2L and SNF2H, contains conserved motifs, KRAR (with lysine corresponding to K72 of S-HDAg) and KVPR (with lysine corresponding to K814 of SNF2L and K799 of SNF2H, respectively), that mimic the native acetylated histone H3/H4 ligand motif (KacXXR) recognized by BAZ2B-BRD (25, 39). To better understand the binding of these motifs to BAZ2B-BRD, we subcloned, expressed, and purified the BRDs of BAZ2B (residues 2054–2168) and its close homolog BAZ2A (residues 1796–1899), and performed isothermal titration calorimetry (ITC) assays using synthetic acetylated peptides.

Initially, ITC assays conducted under standard salt conditions (150 mM NaCl) failed to yield detectable binding signals. Given that structural analyses of resolved BRD–Kac peptide complexes indicate these interactions are often mediated by water-bridged hydrogen bonds alongside hydrophobic packing (39, 40), we repeated the ITC measurements under low-salt conditions (50 mM NaCl). The data revealed that the S-HDAg-K72ac peptide (residues 67–77) binds weakly to both BRDs, with a modestly higher affinity for BAZ2A-BRD (*K*_d_ = 459 μM for BAZ2A-BRD and 1066 μM for BAZ2B-BRD; Fig. 1*B*). These affinities are substantially weaker than those measured for the native histone ligand H3K14ac (residues 1–19), which binds with *K*_d_ values of 18 μM (BAZ2A-BRD) and 5.6 μM (BAZ2B-BRD; Fig. S1, *A* and *B*). ITC assays also confirmed the interaction between BAZ2A/B-BRD and acetylated SNF2L/H peptides (Fig. S1, *C* and *D*), consistent with prior findings (25). Thermodynamic analyses indicated that the interactions between Kac peptides and BAZ2A/B-BRD are enthalpy-driven (Fig. S2), suggesting the involvement of multiple hydrogen bonds facilitated by water molecules (41), in agreement with previous structural studies (39, 40). Although the binding affinities of S-HDAg-K72ac for BAZ2A/B-BRD are in the high micromolar range, this is consistent with the generally weak affinities reported for many acetylated histone–BRD interactions (42).

Finally, a structure-based sequence alignment of BAZ2A-BRD and BAZ2B-BRD shows 59.41% identity (60/101 residues), 26.73% similarity (27/101 residues), and 13.86% difference (14/101 residues), with identical secondary structure organization (Fig. 1*C*) (43, 44). This high degree of conservation supports their related functional roles, as also reflected in our ITC data and previous study (39). In summary, ITC assays demonstrate that the S-HDAg-K72ac peptide binds to both BAZ2A-BRD and BAZ2B-BRD with high micromolar affinity, with BAZ2A-BRD exhibiting a moderately stronger interaction.

### Structural basis for the recognition of acetylated S-HDAg-K72 by BAZ2A-BRD

**Figure 2:**
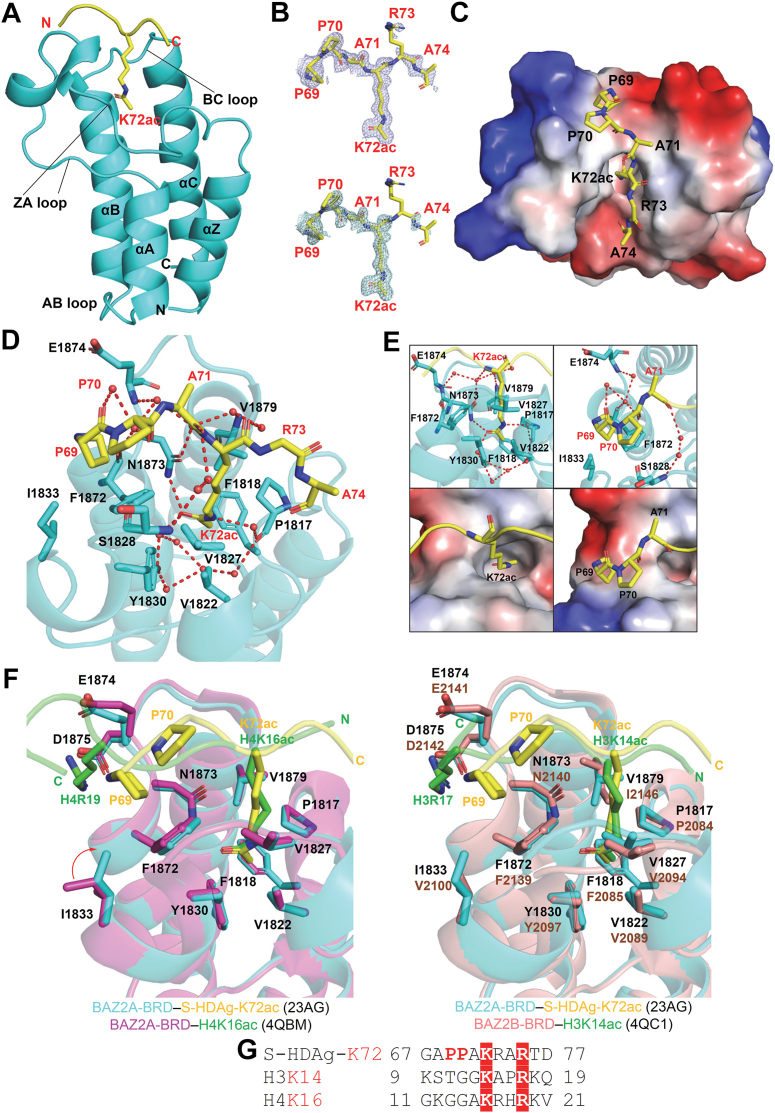
**Structural basis for S-HDAg-K72ac recognition by the BAZ2A BRD.***A*, overall structure of BAZ2A-BRD in complex with S-HDAg-K72ac peptide. BAZ2A-BRD is shown in *cyan* (cartoon) and the peptide in *yellow* (cartoon). *B*, electron density maps of the S-HDAg–K72ac peptide. ${2F}_{o}-F_{c}$ electron density map (*bluemesh*, *upper panel*) is contoured at 1.0*σ*, showing continuous density for the peptide backbone from residues P69 to K72. ${\;F}_{o}-F_{c}$ omit map (*greenmesh*, *lower panel*), calculated after omission of the peptide, is contoured at 3.0*σ* and confirms the presence of the peptide, including the acetylated Lys72 (K72ac). Key residues are labeled for clarity. Maps were generated using PHENIX/PyMOL. *C*, electrostatic potential (isocontour value of ± 83 *kT*/*e*; *red*, negative; *blue*, positive) surface representation of the BAZ2A-BRD–S-HDAg-K72ac complex. The peptide is shown in stick representation. The side chain of S-HDAg-R73 is omitted due to insufficient electron density. *D*, detailed interactions between the S-HDAg-K72ac peptide and BAZ2A-BRD. Key residues are shown as sticks, and intermolecular hydrogen bonds are indicated by *reddashed lines*. *E*, key residue interactions in the BAZ2A-BRD–S-HDAg-K72ac interface. S-HDAg and BAZ2A residues are labeled in *red* and *black*, respectively. *F*, superposition of the BAZ2A-BRD–S-HDAg-K72ac (PDB: 23AG, this work) complex onto the BAZ2A-BRD–H4K16ac (PDB: 4QBM, *left*) and BAZ2B-BRD–H3K14ac (PDB: 4QC1, *right*) complexes, respectively. *G*, sequence alignment of the S-HDAg-K72, H3K14, and H4K16 peptides. The conserved acetylated lysine (K) and the arginine (R) at the Kac+3 position are highlighted with a *red* background. Residues of S-HDAg-K72ac involved in interactions are labeled in *red*. Structure figures were generated using PyMOL. Electrostatic potential surfaces were calculated with PyMOL’s built-in protein contact potential function, with negatively and positively charged regions shown in *red* and *blue*, respectively (61).

To elucidate the molecular mechanism underlying the interaction between S-HDAg-K72ac and the BRDs of BAZ2A and BAZ2B, we attempted to crystallize these BRDs in complex with the S-HDAg-K72ac peptide. We successfully solved the crystal structure of the BAZ2A-BRD–S-HDAg-K72ac complex (Fig. 2 and Table 1).

**Table 1:** Data collection and refinement statistics

Complex	BAZ2A-BRD–S-HDAg-K72ac
PDB code	23AG
Data collection	
Space group	P3_1_21
Cell dimensions	
*a, b, c* (Å)	95.4, 95.4, 33.2
*α, β, γ* (◦)	90, 90, 120
Resolution (Å)	82.64∼1.61 (1.64∼1.61)
Measured reflections	43,035 (1849)
Unique reflections	22,413 (984)
*R*_merge_	0.049 (0.407)
*I/σI*	10.4 (2.0)
CC_1/2_	0.994 (0.642)
Completeness (%)	98.7 (87.5)
Redundancy	14.5 (4.9)
Model refinement	
Resolution (Å)	82.64∼1.61 (1.67∼1.61)
*R*_*work*_/*R*_*free*_ (%)	18.5/20.4
No. atoms/average *B*-factors (Å^2^)	1071/26.6
Protein	885/25.0
Ligand	12/23.8
Water	174/35.4
Root mean square deviation	
Bond lengths (Å)	0.010
Bond angles (°)	0.82
Ramachandran plot % residues	
Favored	98.02
Additional allowed	1.98
Disallowed	0.00

Values in parentheses are for the highest resolution shell.

In the complex structure, BAZ2A-BRD adopts the canonical four-α-helix bundle (αZ, αA, αB, αC) connected by three inter-helical loops (ZA loop, AB loop, BC loop; Fig. 2*A*), as described previously (39, 40, 45). The S-HDAg-K72ac peptide, traced with electron density for residues P69 to K72, binds to the conserved acetyl-lysine binding pocket, similar to complexes reported in prior structural studies (Fig. 2, *A* and *C*) (39, 40). The ${2F}_{o}-F_{c}$ electron density map, contoured at 1.0*σ*, shows continuous density for the peptide backbone from residues P69 to K72, including the region surrounding K72ac (Fig. 2*B*, *upper* panel). The composite ${\;F}_{o}-F_{c}$ omit map, calculated after removal of the peptide and contoured at 3.0*σ*, reveals positive density consistent with the modeled peptide trajectory (Fig. 2*B*, *lower* panel). Weak but characteristic density corresponding to the di-proline motif and the acetylated K72 supports both the sequence register and the assigned orientation of the peptide. Detailed interactions include: The side chain of K72ac inserts into a hydrophobic pocket formed by P1817, F1818, V1822, V1827, Y1830, F1872, and V1879 of BAZ2A-BRD, forming multiple direct or water-mediated hydrogen bonds with the side chains of Y1830 and N1873 and the main chain of P1817. Residues P69 and P70 of the peptide further stabilize the interaction *via* water-bridged hydrogen bonds between their backbone carbonyl groups and F1872/E1874 of BAZ2A-BRD, alongside hydrophobic packing of their side chains with I1833 and F1872 (Fig. 2, *C–E*).

Unexpectedly, structural comparison with previously solved BAZ2A-BRD–H4K16ac (PDB: 4QBM) and BAZ2B-BRD–H3K14ac (PDB: 4QC1) complexes revealed that the S-HDAg-K72ac peptide binds to BAZ2A-BRD in an inverted orientation (Fig. 2*F*). A similar reversal of peptide binding orientation has been reported for the BRDs of PCAF (p300/CBP-associated factor, also known as histone acetyltransferase KAT2B) with two acetylated histone H3 peptides (Fig. S3, *A* and *B*) (46) and of BPTF (bromodomain and PHD finger-containing transcription factor, another regulatory subunit of ISWI chromatin remodeling complexes) with an H4K16ac peptide (Fig. S3, *C* and *D*) (47). Despite the reversed binding, the key BRD residues involved in the interaction superimpose well between the structures. This includes the two critical negatively charged residues, E1874 (corresponding to E2141 in BAZ2B) and D1875 (corresponding to D2142 in BAZ2B), which recognize the signature arginine residue (H4R19 in H4K16ac and H3R17 in H3K14ac) in the KacXXR motif *via* electrostatic interactions (Fig. 2*F*).

S-HDAg was initially proposed as a BAZ2B ligand based on the presence of a KacXXR motif (_72_KacRAR_75_) (Fig. 2*G*) (25). However, in the inverted binding orientation observed in our structure, P69 occupies the position analogous to R75 and engages in a hydrophobic interaction with I1833 of BAZ2A-BRD (Fig. 2, *D* and *E*). This interaction is facilitated by a local conformational adjustment of I1833 relative to its position in the histone peptide complex (Fig. 2*F*). The inverted binding mode of the S-HDAg-K72ac peptide provides a direct structural explanation for its weak binding affinity to BAZ2A/B-BRD observed in our ITC assays (Fig. 1*B*). In summary, structural analysis of the BAZ2A-BRD–S-HDAg-K72ac complex reveals that the viral peptide binds to the BRD in an inverted orientation. This unusual binding mode is consistent with the measured weak affinity and highlights the binding plasticity of BAZ2A/B-BRD.

### Mutation of key interacting residues differentially affects binding

**Figure 3:**
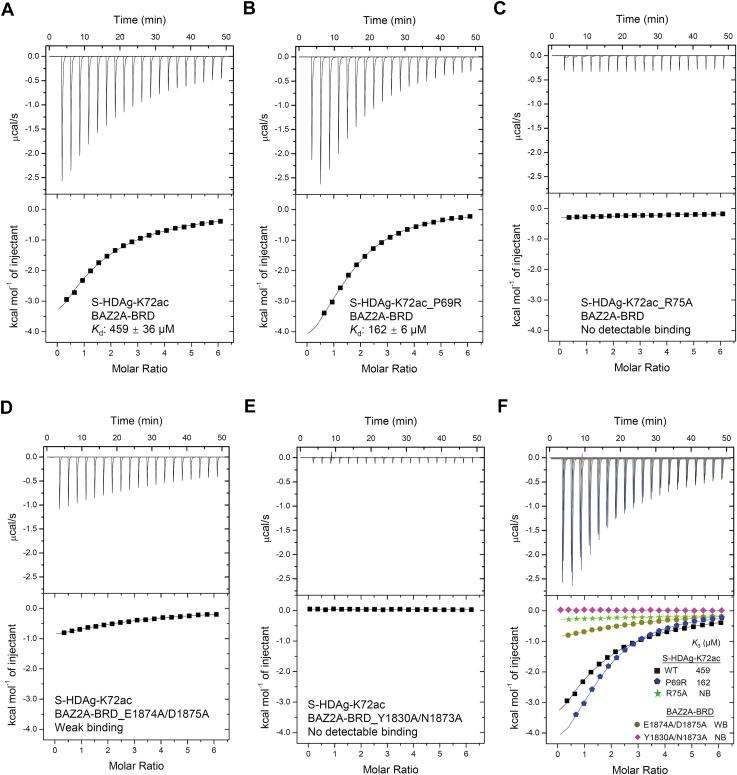
**Mutagenesis reveals critical interaction determinants between BAZ2A-BRD and S-HDAg-K72ac.***A–C*, ITC binding curves for the titration of wild-type S-HDAg-K72ac *A*, S-HDAg-K72ac_P69R *B*, and S-HDAg-K72ac_R75A *C*, mutant peptides into wild-type BAZ2A-BRD protein. *D, E*, ITC binding curves for the titration of wild-type S-HDAg-K72ac peptide into the BAZ2A-BRD_E1874A/D1875A *D*, BAZ2A-BRD_Y1830A/N1873A *E* mutants. *F*, superimposed ITC titration curves from panels *A*–*E*. *K*_d_: dissociation constant (μM); NB, no detectable binding; WB, weak binding.

**Figure 4:**
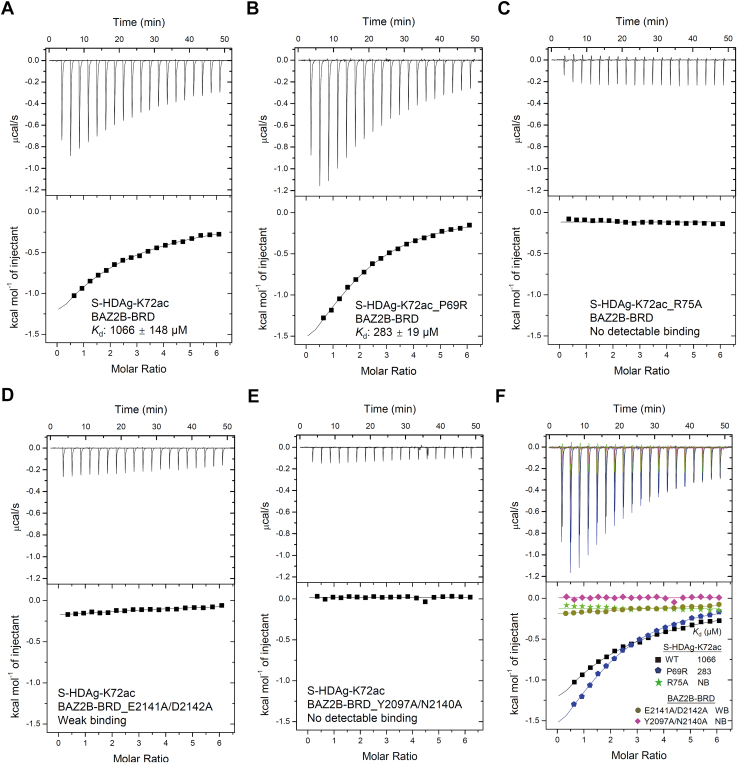
**Mutagenesis reveals critical interaction determinants between BAZ2B-BRD and S-HDAg-K72ac.***A–C,* ITC binding curves for the titration of wild-type S-HDAg-K72ac *A*, S-HDAg-K72ac_P69R *B*, and S-HDAg-K72ac_R75A *C*, mutant peptides into wild-type BAZ2B-BRD protein. *D* and *E*, ITC binding curves for the titration of wild-type S-HDAg-K72ac peptide into the BAZ2B-BRD_E2141A/D2142A *D*, BAZ2B-BRD_Y2097A/N2140A *E*, mutants. *F*, superimposed ITC titration curves from panels *A*–*E*. *K*_d_: dissociation constant (μM); NB, no detectable binding; WB, weak binding.

To validate the findings from our crystal structure analysis, we performed binding assays using S-HDAg-K72ac peptides containing specific point mutations. Replacing P69 with an arginine (P69R) significantly enhances the interaction with both BRDs (*K*_d_ = 162 μM for BAZ2A-BRD vs. 459 μM for the wild-type peptide; *K*_d_ = 283 μM for BAZ2B-BRD vs. 1066 μM for the wild-type peptide; Figs. 3, *A*, *B*, *F* and 4, *A*, *B*, *F*). This result is consistent with the inverted binding orientation observed in our structure and aligns with the known preference of these BRDs for a KacXXR motif (39). In contrast, the R75A mutation abolishes the interaction of S-HDAg-K72ac with both BRDs (Figs. 3, *C*, *F* and 4, *C*, *F*). Although the electron density for residue R75 was not well defined in the structure, it is positioned near a negatively charged surface (Fig. 2*C*). The R75A mutation likely disrupts critical charge–charge interactions that facilitate initial binding, thereby blocking binding, consistent with previous studies (48, 49). An alternative explanation is that a dual-orientation equilibrium may exist in solution, as supported by studies on BPTF-BRD (47). This possibility is corroborated by mutagenesis of the BRDs themselves: Mutations of the negatively charged residues critical for recognizing the arginine in the KacXXR motif, BAZ2A-BRD_E1874A/D1875A, and BAZ2B-BRD_E2141A/D2142A, significantly weaken the interaction (Figs. 3, *D*, *F* and 4, *D*, *F*). Additionally, recognition of the acetylated lysine is essential for binding, as confirmed by mutations in two key residues involved in Kac coordination: BAZ2A-BRD_Y1830A/N1873A and BAZ2B-BRD_Y2097A/N2140A. These mutants completely disrupt the interaction (Figs. 3, *E*, *F* and 4, *E*, *F*).

Taken together, our mutagenesis data confirm that the BRDs of BAZ2A and BAZ2B engage the S-HDAg-K72ac peptide with a preference for the inverted binding orientation observed in the crystal structure. The critical role of R75, despite its absence from the electron density, raises the possibility that the peptide may sample additional binding modes in solution, such as the canonical orientation seen with histone ligands (Fig. 2*F*). A dual-orientation equilibrium in solution may also apply to the BAZ2 BRDs, as previously observed for the BPTF-BRD–H4K16ac complex (Fig. S3, *C* and *D*) (47). However, direct evidence for such alternative modes in the BAZ2 system will require further investigation.

### Mutations in key S-HDAg residues affect its interaction with BAZ2A/B-BRD in cells

**Figure 5:**
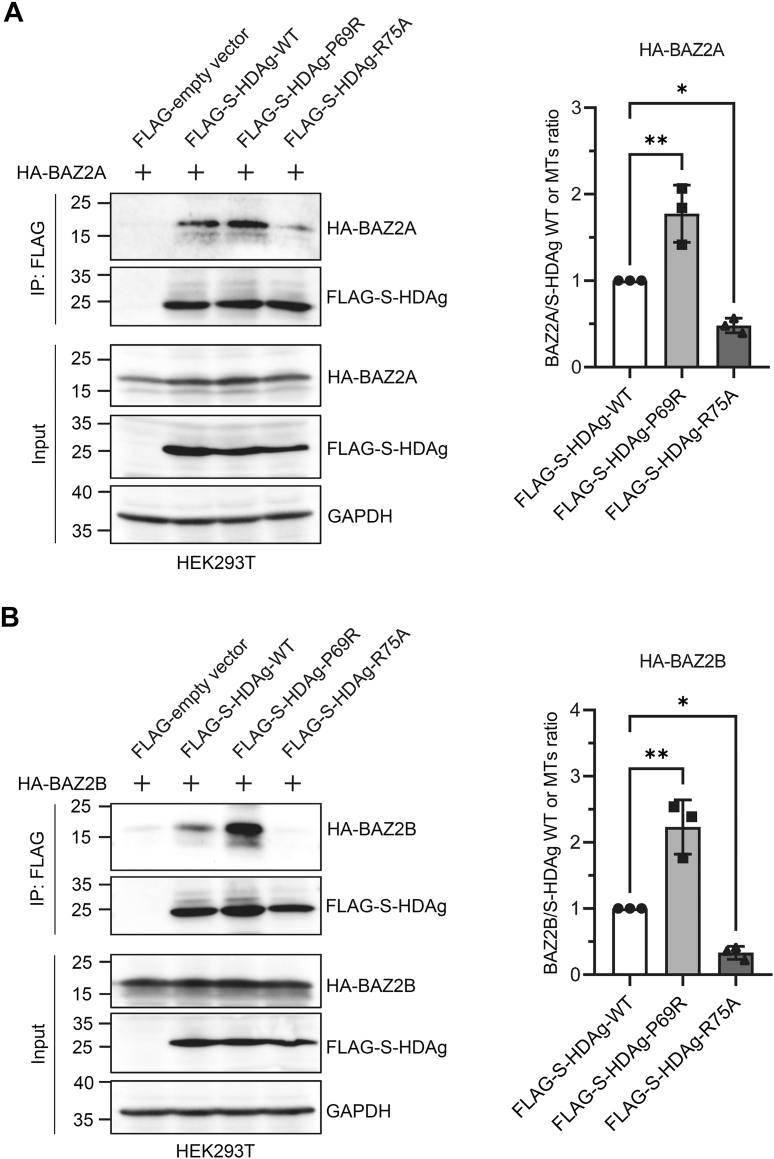
**Mutation of key interacting residues in S-HDAg affects its interaction with BAZ2A/B-BRD in cells.***A and B*, immunoblots of FLAG co-immunoprecipitates from HEK293T cells transfected with 3×FLAG-empty vector or the indicated 3×FLAG-S-HDAg constructs, along with wild-type HA-tagged BAZ2A-BRD *A*, or HA-tagged BAZ2B-BRD *B*. WT: wild-type. Data shown (immunoblot image) are representative of three independent experiments. Statistical analysis data were presented as mean ± SD (n = 3), Student’s *t* test, ∗: *p* < 0.05; ∗∗: *p* < 0.01.

To evaluate the functional importance of critical S-HDAg residues for its interaction with BAZ2A/B-BRD in cells, we co-expressed HA-tagged wild-type BAZ2A-BRD or BAZ2B-BRD with FLAG-tagged wild-type or mutant S-HDAg in HEK293T cells. Co-immunoprecipitation (co-IP) using anti-FLAG affinity gel showed that wild-type S-HDAg efficiently co-precipitates BAZ2A-BRD and BAZ2B-BRD (Fig. 5, *A* and *B*). The S-HDAg-P69R mutant markedly enhances this interaction, whereas the S-HDAg-R75A mutant severely impairs it (Fig. 5, *A* and *B*), in agreement with the previous report with R75A weakening the interaction (25). These cellular results are consistent with our structural and biophysical data, further supporting the conclusion that the BRDs of BAZ2A and BAZ2B favor an inverted binding mode for the S-HDAg-K72ac peptide, while both possible orientations may contribute to the interaction.

Binding of the S-HDAg-K72ac peptide to BAZ2A/B-BRD *in* *vitro* occurs with modest micromolar to near-millimolar affinities (*K*_d_ = 459–1066 μM). However, during viral replication, the functional interaction between S-HDAg and the host chromatin-remodeling machinery is likely cooperatively strengthened by additional contact surfaces beyond the K72ac hot spot. Consistent with this view, a recent NMR study by Yang *et al.* solved the structure of the C-terminal S^Δ60^ domain (residues 56–195) of S-HDAg (50). Importantly, that work identified a conserved cluster of glutamic acid residues (E125–E129) on the surface of S^Δ60^ that is critical for HDV replication. The authors proposed that this surface acidic patch may function by mimicking the nucleosome acidic patch, a well-established hub for the recruitment of multiple chromatin-remodeling complexes, including the ISWI family remodelers BAZ2A/B-containing BRF complexes (51, 52, 53). In line with this hypothesis, the acidic surface of S^Δ60^ was found to be essential for viral replication (50).

**Figure 6:**
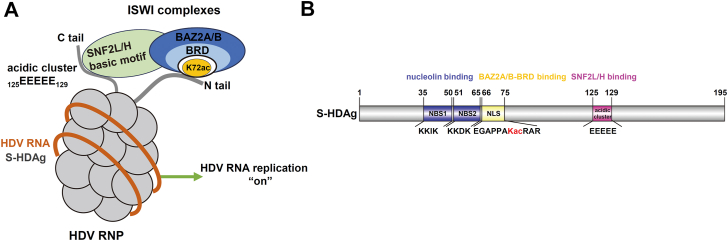
**Proposed model for the recruitment of ISWI chromatin remodeler complexes by S-HDAg during HDV replication.***A*, a synergistic dual-interface model for S-HDAg-mediated recruitment of BAZ2A/B chromatin remodelers. *B*, schematic representation of the domain architecture of S-HDAg, highlighting functional motifs and their known interacting partners. NBS1 (residues 35–50, core motif _39_KKIK_42_) and NBS2 (residues 51–65, core motif _60_KKDK_63_) comprise the nucleolin binding sites. The NLS motif (residues 65–75) contains K72ac, which mediates binding to the BAZ2A/B bromodomains. An acidic cluster (residues 125–129) is proposed to engage SNF2L/H.

Building on these findings, we propose a synergistic dual-interface model for S-HDAg-mediated recruitment of BAZ2A/B chromatin remodelers during HDV replication (Fig. 6*A*). In this model, which can be tested in future studies, the N-terminal K72ac linear motif serves as a specific recognition element for the BAZ2A/B bromodomains, initiating the recruitment process. The C-terminal acidic cluster on S^Δ60^ then provides a complementary interaction surface, potentially engaging the same remodeler complex, perhaps through the conserved basic motif within the catalytic subunit of ISWI remodelers, SNF2L/H (51), to stabilize the overall assembly and promote efficient HDV RNA replication. This model is analogous to how many chromatin-associated factors utilize both a specific modification-binding domain and an adjacent acidic surface to achieve high-avidity and processive engagement with their target complexes. Moreover, the current model of HDV ribonucleoproteins suggests that HDV RNA wraps around HDAg multimers in a manner reminiscent of DNA condensation with histones and may form nucleosome-like structures (25, 54). The low degree of polymerization of S-HDAg may generate multivalent binding, which would enhance the overall affinity. Thus, our structural and biophysical characterization of the K72ac–BAZ2-BRD interaction, combined with the discovery of a functionally essential acidic patch in S^Δ60^, provides an integrated molecular framework for how HDV hijacks the host ISWI chromatin-remodeling machinery by presenting two distinct surface features on a single viral antigen.

Another compelling example supporting the multidomain interaction model comes from the essential interaction between S-HDAg and the multifunctional nucleolar phosphoprotein nucleolin (55). Although nucleolin is not a component of the BAZ2A/B chromatin-remodeling complex, its fundamental role in regulating ribosome biogenesis and chromatin structure makes it a relevant host target. Lee and colleagues established that the N-terminal domain (residues 35–65) of S-HDAg binds directly to the nucleolar protein nucleolin *via* two highly conserved K(K/R)XK motifs. Importantly, functional analyses revealed that a mutated S-HDAg lacking the second N-terminal nucleolin binding site (NBS2, residues 51–65, core motif K(K/R)XK) completely fails to support HDV replication (55), demonstrating the critical nature of this interaction. Strikingly, this K(K/R)XK motif is positioned within the N-terminal region, immediately preceding the KacXXR motif (residues 67–77) we have characterized for BAZ2A/B BRD binding (Fig. 6*B*). This tight clustering of distinct, essential interaction modules within the first ∼80 amino acids of S-HDAg is highly instructive. It suggests a model in which the N-terminus of S-HDAg acts as a small but dense molecular hub, where cooperative binding of multiple host factors *via* adjacent, moderate-affinity interfaces collectively achieves the functional specificity and avidity required for productive viral replication.

Taken together, our work, combined with the existing literature on nucleolin (55) and the recent structural work on the C-terminal S^Δ60^ domain (50), refines the model of S-HDAg function. The picture emerges that S-HDAg is not a simple one-hit binder. Instead, it acts as a multivalent signaling hub, deploying distinct, short, and moderate-affinity motifs across its structure to cooperatively engage crucial host factors, thereby orchestrating a stable functional complex for HDV replication in the nucleus.

In conclusion, our study provides the structural basis by which HDV co-opts host chromatin remodelers *via* an atypical, weak-affinity interaction. We clearly demonstrate that the acetylated viral peptide S-HDAg-K72ac binds directly to the BRDs of BAZ2A and BAZ2B with high micromolar affinity, with BAZ2A-BRD exhibiting moderately stronger binding. The crystal structure of the BAZ2A-BRD–S-HDAg-K72ac complex reveals an unexpected inverted binding orientation of the peptide compared to canonical histone ligands, directly explaining the weak interaction. Mutagenesis, biophysical assays, and cellular co-immunoprecipitation experiments confirm that these BRDs display a preference for this inverted mode, while both possible orientations may contribute to the interaction. Collectively, these findings uncover a novel molecular strategy employed by HDV to hijack host epigenetic machinery and expand our understanding of the binding plasticity within the BAZ2 bromodomain family. Our work thus represents a significant advance in the molecular virology of HDV and provides a structural foundation for future therapeutic targeting.

## Experimental procedures

### Protein expression and purification

The cDNA sequences encoding the BRDs of BAZ2A (residues 1796–1899) and BAZ2B (residues 2054–2168) were synthesized by Azenta Life Sciences with codon optimization for expression in *E. coli*. Each cDNA fragment was subcloned into a modified pET28-MHL vector *via* *Nde*I and *Hind*III restriction sites. This generated constructs for expressing recombinant proteins fused to an N-terminal 6×His tag followed by a tobacco etch virus (TEV) protease cleavage site.

Recombinant proteins were overexpressed in *E. coli* BL21 (DE3) Codon Plus RIL cells (TransGen, CD601). Cells were grown in Luria-Bertani medium at 37 °C to an OD_600_ of 0.6 to 0.8, cooled, and protein expression was induced with 0.25 mM isopropyl-β-D-thiogalactopyranoside (IPTG), followed by incubation at 15 °C for 24 h. Cells were harvested by centrifugation (6000 rpm, 4 °C, 10 min) and resuspended in lysis buffer (20 mM Tris-HCl, pH 7.5, 500 mM NaCl, 5% glycerol). Lysates were prepared by sonication (25% power, 3-s on/7-s off, 15 min, 2–3 cycles) and clarified by centrifugation (11,000 rpm, 4 °C, 50 min). The supernatant was loaded onto a Ni^2+^-nitrilotriacetate affinity column (GE Healthcare, 17526802). Bound proteins were washed and eluted under the following conditions: wash buffer (20 mM Tris-HCl, pH 7.5, 1 M NaCl, 40 mM imidazole); elution buffer (20 mM Tris-HCl, pH 7.5, 250 mM NaCl, 250 mM imidazole). The eluted protein was treated with TEV protease at 4 °C to remove the His-tag. The cleavage mixture was reapplied to the Ni^2+^ column to remove the His-tag fragment, any uncut protein and His-tagged TEV protease. The flow-through containing the untagged protein was further purified by Superdex 75 gel filtration (GE Healthcare, 28989333) with a buffer of 20 mM Tris-HCl, pH 7.5, 150 mM NaCl, 1 mM DTT. Final proteins were concentrated to 15 mg/ml using Amicon Ultra-15 centrifugal filters (Millipore, UFC901024) for ITC and crystallization assays.

All point mutants were generated using the Fast Mutagenesis System Kit (Transgene, FM111-02) according to the manufacturer’s instructions and verified by Sanger sequencing (Azenta Life Sciences). Mutant proteins were expressed and purified using the same protocol as for the wild-type proteins.

### Isothermal titration calorimetry (ITC) assay

Binding affinities were measured using an iTC200 microcalorimeter (MicroCal, Inc.). Lyophilized peptides (synthesized by Shanghai Apeptide Co., Ltd) were dissolved in ITC buffer (20 mM Tris-HCl, pH 7.5, 50 mM NaCl), with pH adjusted by dropwise addition of 2 M NaOH. The peptides used were: S-HDAg-K72ac (residues 67–77, K72 acetylated), H3K14ac (residues 1–19, K14 acetylated), SNF2L-K814ac (residues 809–819, K814 acetylated), and SNF2H-K799ac (residues 794–804, K799 acetylated). The sequence of SNF2L residues 809 to 819 (KTIGYKVPRNP) is identical to that of SNF2H residues 794 to 804. Proteins were diluted into the same buffer.

Protein solutions (200 μM, 50 μM, or 100 μM) in the sample cell were titrated with peptide solutions (6 mM for S-HDAg-K72ac, 0.75 mM for H3K14ac, 1.5 mM for SNF2L-K814ac/SNF2H-K799ac) *via* 20 consecutive injections at 150 s intervals. All the measurements were conducted in duplicate at 25 °C, utilizing an iTC200 microcalorimeter (MicroCal, Inc.). Control titrations of peptide into buffer were performed under identical conditions. Data were processed using the single-site binding model in Origin software (MicroCal, Inc.) after subtracting control heat changes with the fitting error reported.

### Protein crystallization

For crystallization, purified BAZ2A/B-BRD (15 mg/ml) was mixed with the S-HDAg-K72ac peptide at a 1:3 molar ratio. Crystals were grown at 18 °C using the sitting-drop vapor diffusion method by mixing 0.5 μl of the protein–peptide mixture with 0.5 μl of reservoir solution. The BAZ2A-BRD–S-HDAg-K72ac complex crystallized in 0.1 M Tris-HCl, pH 8.5, 25% w/v polyethylene glycol 3350. Crystals were briefly transferred to a cryoprotectant solution (85% reservoir solution, 15% glycerol) before flash-freezing in liquid nitrogen.

### Data collection and structure determination

X-ray diffraction data were collected at 100 K on beamline BL10U2 at the Shanghai Synchrotron Radiation Facility (SSRF). Data were processed automatically using the Aquarium pipeline (Porpoise_DIALS) (56), which is based on DIALS (57). The structure was solved by molecular replacement with PHASER (58) using PDB entry 4QBM (39) as the search model. Automated model building and iterative refinement were performed with PHENIX (59). Manual model inspection and adjustment were carried out in Coot (60). Data collection and refinement statistics are summarized in Table 1. All structural figures were prepared using PyMOL.

### Cell transfection and FLAG co-immunoprecipitation (co-IP)

HEK293T cells (ATCC, CRL-11268) were obtained from the American Type Culture Collection, which authenticates its human cell lines by short tandem repeat (STR) profiling. The cells were authenticated by STR analysis and tested for *mycoplasma* contamination upon receipt. Routine tests for *mycoplasma* contamination were performed *via* visual inspection for microbial growth and turbidity to ensure that all experiments were conducted with *mycoplasma*-free cells; no visible contamination was observed throughout the study. Cells were cultured in Dulbecco’s modified Eagle’s medium (DMEM; Gibco, C11995500BT, high glucose) supplemented with 10% fetal bovine serum (FBS; Sigma-Aldrich, F0193) and 100 units/ml penicillin/streptomycin (NCM Biotech, C100C5) at 37 °C in a humidified atmosphere containing 5% CO_2_.

HEK293T cells were co-transfected with plasmids encoding 3×FLAG-tagged constructs (empty vector, wild-type S-HDAg, S-HDAg-P69R, or S-HDAg-R75A) and wild-type HA-tagged BAZ2A-BRD or BAZ2B-BRD using polyethyleneimine (PEI, Sigma-Aldrich, P3143). At 48 h after transfection, cells were harvested and lysed in RIPA buffer (50 mM Tris-HCl, pH 7.4, 150 mM NaCl, 1% Triton X-100, 0.1% SDS, 1 mM EDTA, protease inhibitor cocktail). Lysates were clarified by centrifugation (12,000 *g*, 10 min, 4 °C) and incubated with pre-washed anti-FLAG affinity gel (Yeasen, 20584ES08) overnight at 4 °C. The gel was washed three times with RIPA buffer, and bound proteins were eluted twice with FLAG peptide (200 μg/ml, DYKDDDDK, ChinaPeptides). Eluates were combined for downstream analysis.

Proteins were separated by SDS-PAGE and transferred to PVDF membranes (Millipore, IPVH00010). Membranes were blocked with 10% non-fat milk in TBST for 1 h at room temperature, then incubated with primary antibodies overnight at 4 °C: rabbit anti-FLAG (Proteintech, 20543-1-AP, 1:1,000, RRID No. AB_11232216), rabbit anti-HA (Proteintech, 51064-2-AP, 1:1,000, RRID No. AB_11042321), or mouse anti-GAPDH (Proteintech, 60004-1-Ig, 1:10,000, RRID No. AB_2107436). After washing, membranes were incubated with HRP-conjugated secondary antibodies (goat anti-mouse IgG, Jackson ImmunoResearch, 115-035-062, 1:10,000, RRID No. AB_2338504; goat anti-rabbit IgG, Jackson ImmunoResearch, 115-035-045, 1:10,000, RRID No. AB_2337938) for 1 h at room temperature. Signals were detected using a Super ECL chemiluminescent substrate kit (NCM Biotech, P10300) and imaged on a Tanon 5200 system. The co-IP experiments were performed three independent times.

### Statistical analysis

Statistical analyses were performed using GraphPad Prism. Student’s *t* test was utilized to analyze data from two groups. All the data were presented as the means ± standard deviations (SDs), and *p* < 0.05 was considered statistically significant. The significance levels are indicated as follows: ∗: *p* < 0.05, ∗∗: *p* < 0.01.

## Data availability

The coordinates and structure factors of BAZ2A-BRD–S-HDAg-K72ac complex have been deposited in the Protein Data Bank (PDB) with accession code 23AG.

## Supporting information

This article contains supporting information.

## Conflict of interest

The authors declare that they have no conflicts of interest with the contents of this article.
