# Supplementary material for: Structural insights into histone mimicry by the small hepatitis delta antigen
Source: J Biol Chem. 2026 Jun 12;302(8):113252. doi: 10.1016/j.jbc.2026.113252 (PMC13377155; doi:10.1016/j.jbc.2026.113252)
Supplement: Supporting Figures [file mmc1.pdf]

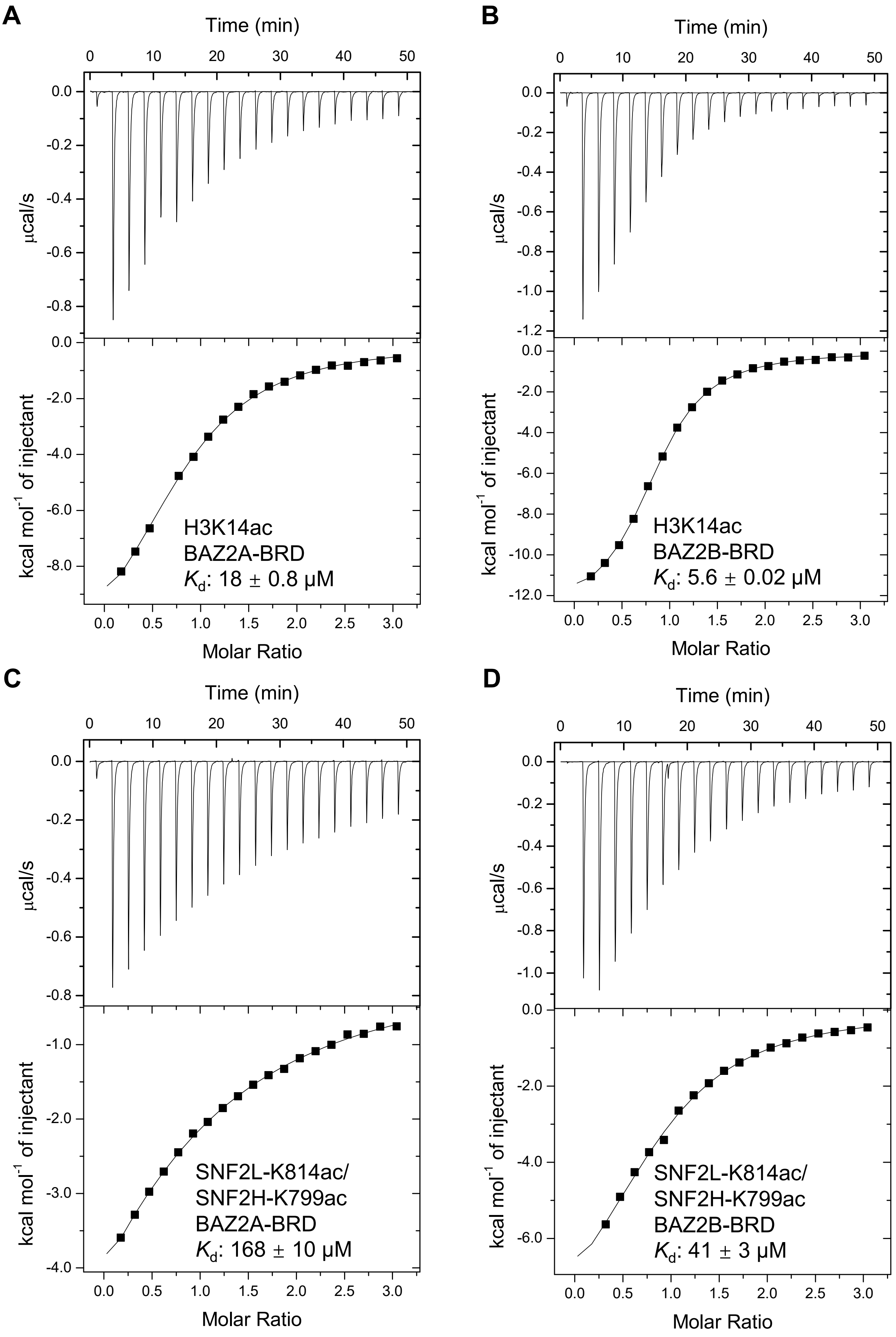

Supplementary Fig. 1 ITC binding curves for the titration of H3K14ac or SNF2L-K814ac/SNF2H-K799ac peptide into BAZ2A/B-BRD proteins. (A, B) Titration of H3K14ac (residues 1–19) peptide into BAZ2A-BRD **(A)** and BAZ2B-BRD **(B)**, respectively. **(C, D)** Titration of SNF2L-K814ac (residues 809–819) or SNF2H-K799ac (residues 794–804) peptide into BAZ2A-BRD **(C)** and BAZ2B-BRD **(D)**, respectively. The sequence of SNF2L residues 809–819 (KTIGYKVPRNP) is identical to that of SNF2H residues 794–804. ITC data shown are representative of two independent experiments performed by an iTC200 microcalorimeter (MicroCal, Inc.). K_d: dissociation constant (μM).

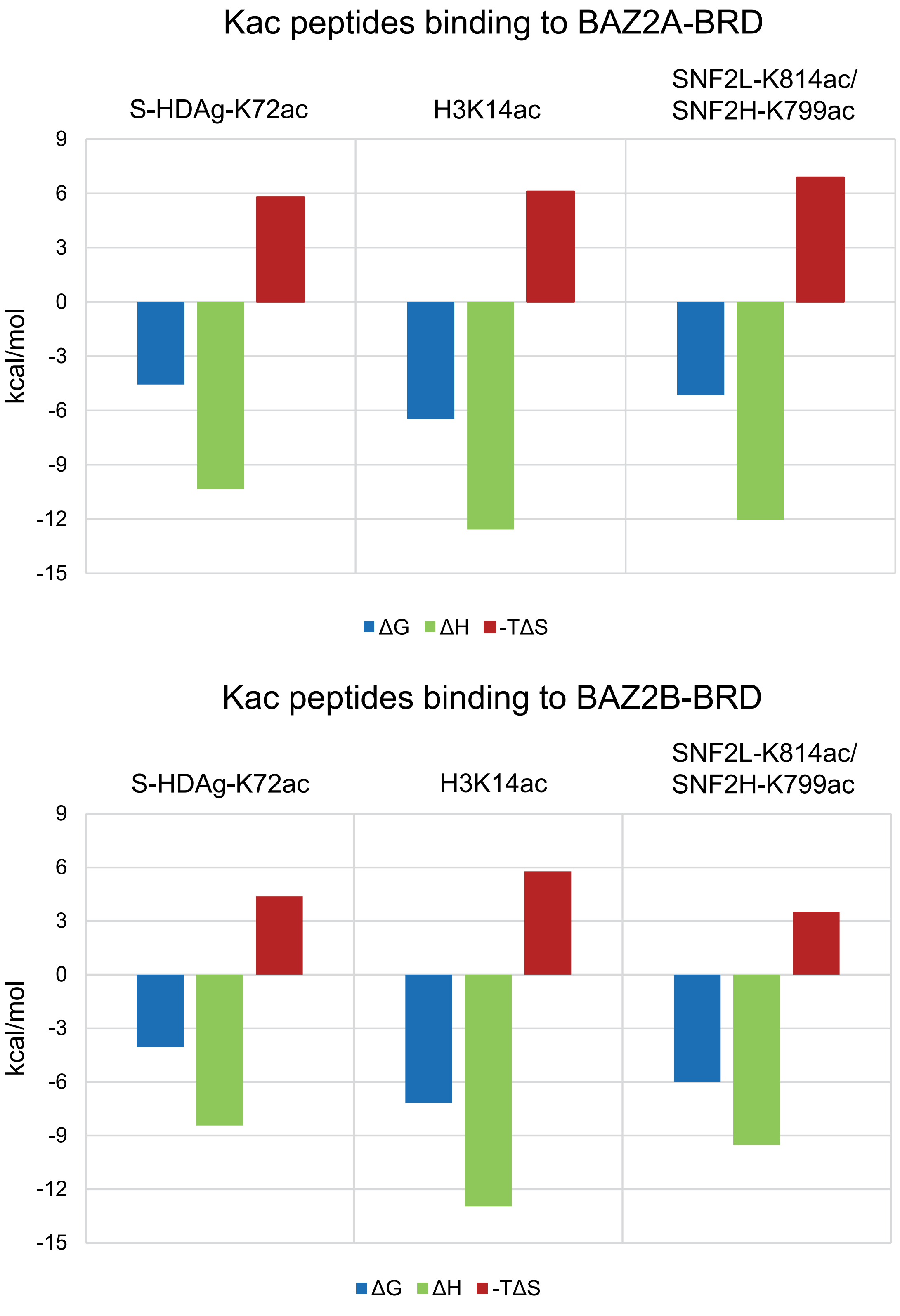

Supplementary Fig. 2 Thermodynamic profiles of Kac peptides binding to the BRDs of BAZ2A (upper panel) and BAZ2B (lower panel), respectively.

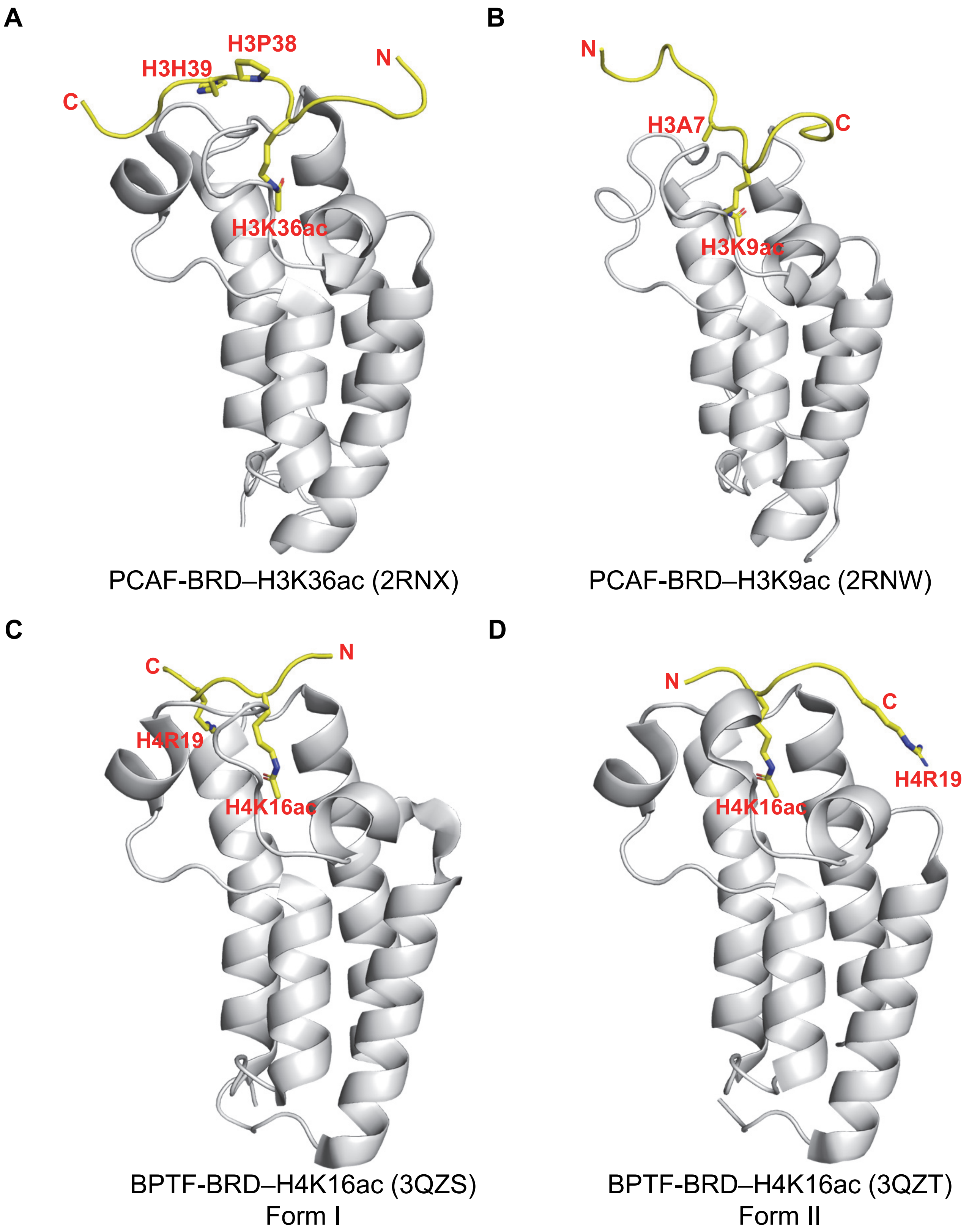

Supplementary Fig. 3 Acetylated histone peptides bind to the same BRD in different orientations. (A, B) Overall structure of PCAF-BRD in complex with H3K36ac **(A)** and H3K9ac **(B)** peptide, respectively. **(C, D)** Overall structure of BPTF-BRD in complex with H4K16ac peptide, showing Form I **(C)** and Form II **(D)**, respectively. The BRD proteins are shown in gray (cartoon) and the acetylated histone peptides in yellow (cartoon). Critical interacting residues of the peptides are shown as sticks.
